# Syn-Lethality: An Integrative Knowledge Base of Synthetic Lethality towards Discovery of Selective Anticancer Therapies

**DOI:** 10.1155/2014/196034

**Published:** 2014-04-22

**Authors:** Xue-juan Li, Shital K. Mishra, Min Wu, Fan Zhang, Jie Zheng

**Affiliations:** ^1^Bioinformatics Research Centre (BIRC), School of Computer Engineering, Nanyang Technological University, 50 Nanyang Avenue, Singapore 639798; ^2^Institute for Infocomm Research (I2R), 1 Fusionopolis Way, Singapore 138632; ^3^Genome Institute of Singapore (GIS), Biopolis, Singapore 138672

## Abstract

Synthetic lethality (SL) is a novel strategy for anticancer therapies, whereby mutations of two genes will kill a cell but mutation of a single gene will not. Therefore, a cancer-specific mutation combined with a drug-induced mutation, if they have SL interactions, will selectively kill cancer cells. While numerous SL interactions have been identified in yeast, only a few have been known in human. There is a pressing need to systematically discover and understand SL interactions specific to human cancer. In this paper, we present Syn-Lethality, the first integrative knowledge base of SL that is dedicated to human cancer. It integrates experimentally discovered and verified human SL gene pairs into a network, associated with annotations of gene function, pathway, and molecular mechanisms. It also includes yeast SL genes from high-throughput screenings which are mapped to orthologous human genes. Such an integrative knowledge base, organized as a relational database with user interface for searching and network visualization, will greatly expedite the discovery of novel anticancer drug targets based on synthetic lethality interactions. The database can be downloaded as a stand-alone Java application.

## 1. Introduction

Finding effective anticancer therapies is a major goal of biomedical research. As a devastating human disease, cancer kills millions of people each year. In 2008, the World Health Organization (WHO) predicted that, if new anticancer treatments are not discovered, there will be 26.4 million cancer patients around the world and 17 million cancer deaths by 2030 [1]. The currently prevalent anticancer treatments, chemotherapies, have several limitations, including the drug resistance and the side-effects of toxicity [2]. Although targeted therapies are being developed, the lack of selectivity (i.e., killing both tumour and healthy cells) remains a major issue for current anticancer therapeutics.

**Figure 1:**
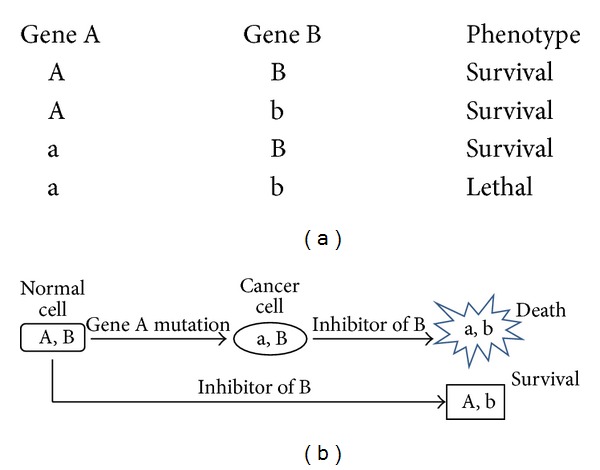
The concept of synthetic lethality. (a) If just one of the SL pair genes is mutated, then the cell is alive. A/B wild type, a/b-mutated genes; (b) mutation/inhibition of one gene or both genes of a SL gene pair leads to different cell fates [2].

Recently, synthetic lethality (SL) has emerged as a novel anticancer strategy that is promising to be highly selective. A pair of genes is defined to have synthetic lethal interactions if the mutation to either gene will not kill the cell but the mutations to both genes will lead to cell death [2] (Figure 1). Compared with healthy cells, cancer cells contain many genetic mutations. Hence, an SL partner of a cancer-specific mutation will be potentially a selective anticancer drug target. A drug that induces a mutation to the SL partner gene will kill cancer cells but spare normal cells, due to the SL interaction with the cancer-specific mutation that is not present in healthy cells.

However, the discovery and clinical applications of SL-based anticancer therapies need to overcome several technical obstacles. Most known SL cases are discovered in yeast, and so far only a few SL gene pairs are known in human. A prevalent technique to discover SL genes is high-throughput screening based on chemical or RNAi libraries [3]. Due to genetic heterogeneity of cancer cells, the SL identified from one screening might not be repeatable in another platform or cancer subtypes. Importantly, the screening-based discovery can hardly yield any mechanistic insight into SL interactions. The interpretation of SL candidates is crucial for reliable application of SL-based therapies. To address these issues, systems biology approaches that can uncover the molecular mechanisms of SL in cancer cells would be needed.

The technique of SL was originated from yeast genetics [4]. Due to its rapid generation time, simple culture, and easy-to-handle genetic manipulation,* S. cerevisiae* has been extensively used to study SL [5]. Computational methods have also been developed to predict and analyze yeast SL [6]. In contrast, there is a dearth of resources (e.g., data, knowledge, or bioinformatics tools) available about SL in human cancer. Recently, some methods have been developed to infer human SL from yeast SL, considering that the genome integrity and cell-cycle related genes from yeast are highly conserved with human and closely related with cancer disease [7]. Massive screening of yeast SL interaction can provide valuable information for SL inference of human cancer. For example, Conde-Pueyo et al. applied the yeast-to-human inference method to obtain potential cancer-related SL target and identified SL partners of cancer-related genes that are drug target [8]. It is highly desirable to integrate data of human cancer SL pairs to reduce the follow-up experimental research in the manageable size.

**Figure 2:**
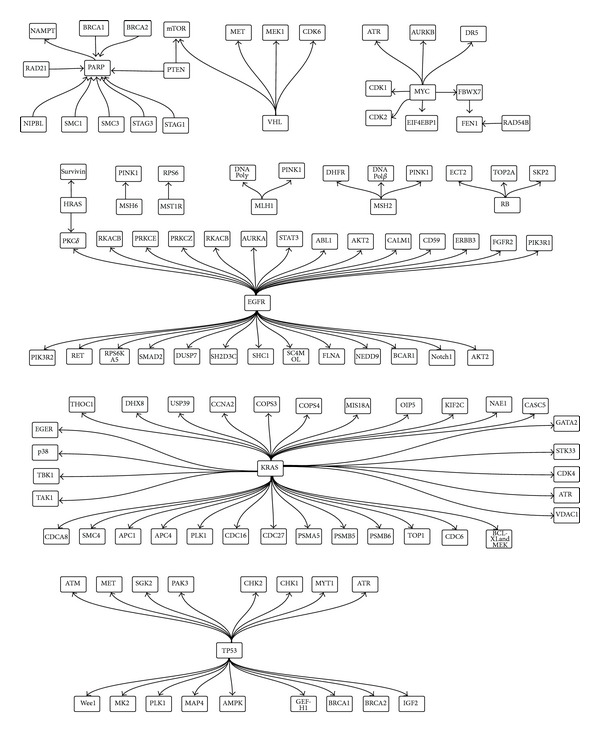
SL network of human cancer constructing based on SL literatures. Each node in the network denotes a gene/protein and each edge represents an SL interaction (the arrow direction leads from mutation gene to target gene).

In this paper, we present an integrative knowledge base dedicated to SL in human cancer, called* Syn-Lethality.* From literature, we collected SL gene pairs that have been experimentally discovered and verified and integrated them into a network (Figure 2), where each node is a gene and each edge represents an SL interaction. We call such a network as* SL network.* Moreover, we associated the SL network with related gene annotations and pathway information, to facilitate mechanistic understanding of SL. In addition to human specific SL, we also collected yeast SL, which were mapped to human genes through orthologous correspondence. The information collected as such has been organized into a relational database with user friendly interface. When users input cancer genes (e.g., TP53), Syn-Lethality will search for SL partners of the query genes and display related annotations (e.g., pathways, gene functions, and hyperlinks to the related literature). The SL network we constructed serves as a roadmap for the whole knowledge base.

To our best knowledge, Syn-Lethality is the first database dedicated to human synthetic lethality. There are few genome wide screenings for SL interactions with human cancer genes, and they are focused on a few well-known oncogenes (e.g., TP53 and KARS). The large-scale screening for human cancer cells is limited by high-cost, false positives, and difficulty to interpret mechanisms, and the information is scattered in the literature. An integrative approach is indispensable for a systematic and mechanistic understanding of human SL. Syn-Lethality database is one of the first attempts to integrate knowledge and data about SL in human cancer. We have also integrated data from yeast and will do so in the future from other model organisms. We believe that it would be a valuable resource and framework that would facilitate novel discovery of potential selective anticancer therapy based on synthetic lethality.

## 2. Data Integration

### 2.1. Data Collection and the Literature Search

The primary aim of our Syn-Lethality database is to collect and maintain a high quality set of SL gene pairs, which serves as a comprehensive, fully classified, and accurately annotated knowledge base for SL-related research. The database also provides extensive cross-references and querying interfaces. The SL pairs in Syn-Lethality database are collected by two alternative methods and we will next introduce them in more detail.

The first method for collecting SL pairs is the literature search. We examined the Web of Knowledge and NCBI PubMed databases with the keywords like “synthetic lethality” and then screened with the keyword “human cancer/tumour” from the abstracts. As such, we collected more than one hundred scientific publications. From these articles, we manually extracted more than one hundred SL gene pairs, which have been verified by experiments for cancer treatment. Although the number of SL pairs collected by the literature search is limited, they are highly trustworthy and thus they lay the foundation for our Syn-Lethality database.

The second source of potential SL pairs is the knowledge transfer from the model organism of yeast to human by comparative genomics analysis. Currently, there are quite a few number of SL pairs in yeast which are experimentally detected by various screening techniques. Meanwhile, some human cancer genes (e.g., related with cell cycle, DNA repair) are observed to be highly evolutionarily conserved with yeast cancer genes for inferring human SL pairs of genes based on human-yeast conservation. Therefore, it is possible to infer some SL pairs in human cancers from yeast. We predict a human gene pair to be an SL pair in human cancer based on the following two constraints. First, this human gene pair has a conserved SL interaction in yeast. Second, one of these two genes is a cancer gene. For example, two yeast genes *y*_*i*_ and *y*_*j*_ form an SL relationship while two human genes *h*_*i*_ and *h*_*j*_ are orthologs of *y*_*i*_ and *y*_*j*_, respectively. If *h*_*i*_ or *h*_*j*_ is a gene that is observed to be mutated in a certain type of cancer, (*h*_*i*_, *h*_*j*_) is then a predicted SL pair in the human cancer. In this paper, all the yeast SL interactions are downloaded from BioGrid [9] (Table 1). However, we noticed that some of these yeast SL pairs from BioGrid involve essential genes. By the definition of SL (i.e., mutation of one gene should not kill the cell, but mutation of both genes kills the cell), both genes in a SL pair should be nonessential. Therefore, with the list of essential genes downloaded from Gerstein Lab at Yale University (http://bioinfo.mbb.yale.edu/genome/yeast/cluster/essential/) and Saccharomyces Genome Deletion Project (http://www-sequence.stanford.edu/group/yeast_deletion_project/) we collected 6,613 SL pairs without any essential genes. In addition, 507 human cancer genes are downloaded from COSMIC: Cancer Gene Census via the link http://cancer.sanger.ac.uk/cancergenome/projects/census/. Finally, we inferred 1,114 SL pairs related with human cancers that are predicted from yeast.

Based on the above* in silico* analysis, the Syn-Lethality database contains 113 SL pairs from NCBI PubMed abstracts and 1,114 SL pairs from the model organism of yeast (Table 3). We also provide additional information about the genes/proteins involved in these SL pairs as shown in Table 1, for example, Entrez gene IDs, full gene name, symbols, gene type (oncogene or tumour suppressor gene), cancer type, pathway information, and some remarks on the molecular mechanisms.

### 2.2. Pathway/Mechanism Analysis of SL Pairs Directly from the Literature

From the list of SL gene pairs, it is interesting to note that a large fraction of SL pairs are involved in fundamental processes of cell fates, cell cycle, and DNA damage response. We first take the KRAS oncogene as an example. Genome-wide RNAi screen was conducted to identify SL interaction partners of KRAS [10]. We observed that the SL interaction partners of KRAS are involved in the mitotic progression, including the subunits of the anaphase-promoting complex/cyclosome (APC/C) complex (ANAPC1, ANAPC4, CDC16, and CDC27), cyclin A2 (CCNA2), kinesin-like protein 2C (KIF2C), KNL-1 (CASC5), hMis18a and hMis18b (C21ORF45 and OIP5), borealin (CDCA8), and SMC4 and polo-like kinase 1 (PLK1). The inhibition of the above genes will lead to the death of cells in which the KRAS has been mutated [10]. TP53 is another example. It is a major downstream effector of DNA-damage kinase pathways. In response to DNA damage, a normal cell will activate a complex signaling network to arrest cell-cycle progression and facilitate the DNA repair. In contrast, TP53-deficient tumor cells rely on other G2/M checkpoint regulators such as checkpoint kinase 1 (CHK1) to arrest cell-cycle progression. Recently, the SL interactions between TP53 (TP53 is mutated) and ATR/Chk1, WEE1, ATM/Chk2, and MK2 targets have been investigated [11]. As an example, myelocytomatosis viral oncogene homolog, MYC, is a multifunctional, nuclear phosphoprotein that plays a role in cell cycle progression, apoptosis, and cellular transformation, as a transcription factor. Overexpression of MYC sensitizes fibroblasts to agonists of the TNF-related apoptosis-inducing ligand (TRAIL) death receptor DR5. It was shown that MYC mediates increased DR5 expression and signaling as a result of enhanced procaspase-8 autocatalytic activities [12].

As reported by [3, 13], the authors proposed the following four types of mechanisms for SL interactions in human cancers from the perspectives of protein complexes and pathways. First, two complexes may be synthetic lethal when they have an essential function in common and they are uniquely redundant. Second, two units within an essential protein complexes may form SL relationship. Third, two components in a linear essential pathway may be SL partners, because the mutation of each component decreases the flow through the pathway but the pathway still has signal flow, whereas the mutation of both will destroy the pathway. Forth, two components in two parallel essential pathways may be backups of each other for the lethality. Generally, the SL pairs can be interpreted as due to the above four mechanisms. For example, EGFR and BRCA1 are SL pairs because they are in the same essential protein complex [14]. In this paper, we will focus on the analysis of SL pairs from the perspective of signalling pathways and provide three SL examples, in which two partners are from two parallel pathways.

First, TANK binding kinase (TBK1) was identified as a synthetic lethal gene of KRAS [15]. TBK1 is a noncanonical inhibitor of B protein (IB) that is known to regulate nuclear factor B (NFB) signalling. TBK1 activates NF-kB antiapoptotic signals involving c-Rel and BCL-XL (also known as BCL2L1) that were essential for survival. These indicate that TBK1 and NF-kB signalling pathways are essential in KRAS mutant tumours. Second, the inhibition of both EGFR and Notch signalling pathway is dramatically more effective for suppressing tumor growth than blocking EGFR or Notch signalling pathway alone. Normally the activated form of Notch1 restores AKT activity and enables cells to overcome cell death after dual-pathway blockade [16]. Here, the combined EGFR and Notch inhibition decreases significantly the AKT activation and thus suppresses tumor growth more effectively. Third, EGFR, a protooncogene, belongs to a family of four transmembrane receptor tyrosine kinases that mediate the growth, differentiation, and survival of cells. It is often overexpressed in aggressive triple negative breast cancers (TNBCs) and is also associated with other aggressive disease phenotypes. Nowsheens group recently reported that a contextual synthetic lethality can be achieved both* in vitro* and* in vivo* with combined EGFR and PARP inhibition with lapatinib and ABT-888, respectively [14]. The mechanism involves a transient deficit of DNA double strand break repair induced by lapatinib and a subsequent activation of the intrinsic pathway of apoptosis. Our Syn-Lethality database contains SL pairs of genes that likely belong to one of the above four mechanisms. The gene function and pathway information in our database will facilitate* in silico* interpretation of mechanisms.

## 3. Database Interface

### 3.1. Usage of SL Database

Our synthetic lethality database contains SL gene pairs in organised form and provides interface to perform query in the database. Our preliminary database is available in the downloadable form from http://www.ntu.edu.sg/home/zhengjie/software/Syn-Lethality/. This software is a Java executable file and requires the installation of Java. The required version 10 of Java (free) and it can be installed from http://www.java.com/en/download/index.jsp. Once the Java is installed on local machine, just double clicking on the Java executable file will launch the database interface. Since the database is available in the single setup file, the database can be used simultaneously by many end users for performing the query (Figure 4). The database includes information such as synthetic lethal gene pairs, type of lethality, type of gene alteration, and target genes for synthetic lethality.

Searching in our database can be divided in the following categories.

**Figure 3:**
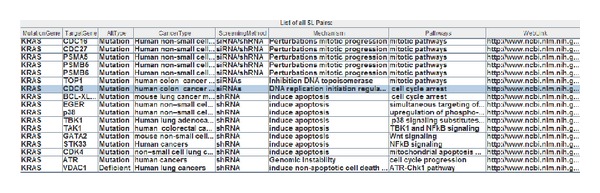
An example of KRAS related SL pairs (the alteration types refer to the cancer mutated gene).

**Figure 4:**
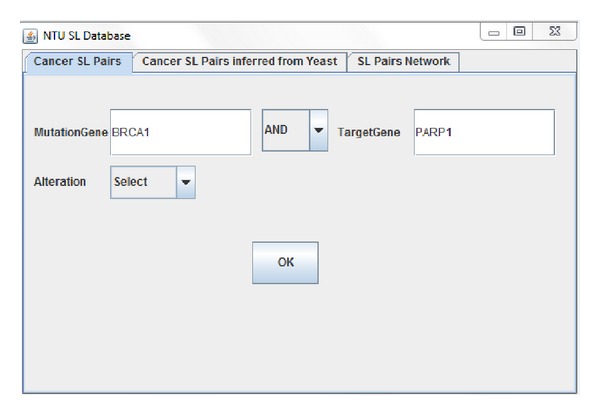
SL query interface.

- *Simple Search*. The user is required to provide abbreviations for gene names. For example, for epidermal growth factor receptor we just need to write EGFR and for cyclin-dependent kinase we just need to write CDK in the search field. This helps the user in search for the SL gene pair information without typing long gene names.
- *Batch Search*. User can directly copy and paste names of various genes (separated by space) in each field.  Figure 3 shows an example of using KRAS as input to query its related SL pairs. This helps find information simultaneously for various synthetic lethal gene pairs.
- *Smart Search*. Users have flexibility of searching SL gene pairs based on the Boolean logical operators by selecting logical AND and OR operators from the drop down menu. This helps in analyzing various combinations of SL gene pairs.
- *Genetic Alteration Search*. The interface of our database provides user flexibility to screen the SL pairs based on various types of the gene alteration which refer to the gene mutated in cancer. The gene alteration types captured in our database includes overexpression, mutation, activation, inactivation, and deficiency.

As of now, it is possible to retrieve complete SL gene pair information based on information such as gene names (MYC, EGFR, CDK, and so forth) (Table 2) and types of genetic alterations (overexpression, mutation, activation, and so forth). The relevant research papers for the SL gene pair are provided via web hyperlinks in database search results.

### 3.2. Synthetic Lethality Network

**Figure 5:**
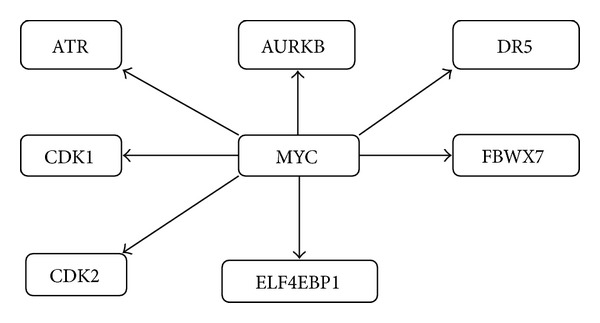
Subnetwork of our SL network for human cancer.

To provide more clear understanding of SL gene pairs, we constructed the network for available SL gene pairs (Figure 2). The diagram depicts the synthetic lethal genes and the target genes. For example, the SL pair information for MYC oncogenic gene is depicted as shown in Figure 5.

## 4. Conclusion and Future Perspectives

Syn-Lethality is the first comprehensive database constructed through integrating experimentally validated SL pairs of human cancer with the inferred SL pairs from yeast according to the orthologous relation between human and yeast. It is the first attempt to apply the experimentally verified SL pairs to construct a SL network. In the SL network, each node represents a gene/protein and each edge denotes the SL interactions which can be easily linked to the annotation information including gene/protein alteration type, screening method, pathway, mechanism, and the related literature. It is a valuable resource for better understanding SL mechanism in human cancer and developing useful information for anticancer medicine.

Considering that our current database only includes the predicted SL pairs from yeast, it is desirable to collect and predict more SL pairs from other model organisms, such as* Caenorhabditis elegans*, Zebrafish, and mouse. With the progress of SL experimental screening technology, it is believed that more SL interactions are expected to be identified. We will continue to collect and curate SL pairs of genes. Additionally, using our SL database, we plan to develop data mining algorithms to quickly extract SL information and mechanistic insights. Moreover, by incorporating the signalling pathways associated with the SL pairs of genes, we will construct a comprehensive and global SL network about human cancer.

## Figures and Tables

**Table 1:** Representative entries for human cancer Syn-Lethality database.

Representative entries	Contents
SL pairs information	Gene names A and B, SL pairs mechanism, and related pathway
SL pairs annotation	Gene symbol, full name, Entrez ID, KEGG-link
Type of gene alterations	Mutation, activation, inactivation, overexpression, deficient
Type of gene involved in SL pairs	Tumor suppressor gene, oncogene
Data source	Human cancer, inferred from yeast
Cancer type	All kinds of cancer
Screening methods	shRNA, siRNA, anticancer compound
The literature search	PMID

**Table 2:** List of annotation database links in Syn-Lethality database.

Database	URL
Biological General Repository for Interaction Datasets (BioGRID)	http://thebiogrid.org/
Saccharomyces Genome Deletion Project (SGDP)	http://www-sequence.stanford.edu/
Catalogue Of Somatic Mutations In Cancer (COSMIC)	http://cancer.sanger.ac.uk/cancergenome/projects/census
The Gene Ontology (GO)	http://www.geneontology.org/
NCBI-Gene	http://www.ncbi.nlm.nih.gov/gene
Kyoto Encyclopedia of Genes and Genomes (KEGG)	http://www.genome.jp/kegg/pathway.html
HUGO Gene Nomenclature Committee (HGNC)	http://www.genenames.org/

**Table 3:** Total statistics for human cancer Syn-Lethality database.

Content	Number	Comments
SL pairs for human cancer from the literatures	113	NCBI PubMed abstracts
SL pairs for human cancer inferred from yeast	1114	Prediction according the orthologous
Screening methods	3	siRNA, shRNA, anticancer compound screening
Type of gene alteration	5	Mutation, deficient, inactivation, activation, overexpression
Annotated human genes	647	Only one gene of SL pairs related to human cancer also counted
Annotated signalling pathways of human genes	647	KEGG pathways

## References

[1] Boyle P, Levin BE (2008). *World Cancer Report*.

[2] Kaelin WG (2009). Synthetic lethality: a framework for the development of wiser cancer therapeutics. *Genome Medicine*.

[3] Kaelin WG (2005). The concept of synthetic lethality in the context of anticancer therapy. *Nature Reviews Cancer*.

[4] Hartwell LH, Szankasi P, Roberts CJ, Murray AW, Friend SH (1997). Integrating genetic approaches into the discovery of anticancer drugs. *Science*.

[5] Heiskanen MA, Aittokallio T (2012). Mining high-throughput screens for cancer drug targetslessons from yeast chemical-genomic profiling and synthetic lethality. *WIREs Data Mining Knowl Discovery*.

[6] Wu M, Li XJ, Zhang F, Li XL, Kwoh CK, Zheng J (2013). *Meta-Analysis of Genomic and Proteomic Features To Predict Synthetic Lethality of Yeast and Human Cancer*.

[7] Yuen KWY, Warren CD, Chen O, Kwok T, Hieter P, Spencer FA (2007). Systematic genome instability screens in yeast and their potential relevance to cancer. *Proceedings of the National Academy of Sciences of the United States of America*.

[8] Conde-Pueyo N, Munteanu A, Solé RV, Rodríguez-Caso C (2009). Human synthetic lethal inference as potential anti-cancer target gene detection. *BMC Systems Biology*.

[9] Chatr-Aryamontri A, Breitkreutz BJ, Heinicke S (2013). The biogrid interaction database: 2013 update. *Nucleic Acids Research*.

[10] Luo J, Emanuele MJ, Li D (2009). A genome-wide RNAi screen identifies multiple synthetic lethal interactions with the ras oncogene. *Cell*.

[11] Morandell S, Yaffe MB (2012). Exploiting synthetic lethal interactions between dna damage signaling, checkpoint control, and p53 for targeted cancer therapy. *Progress in Molecular Biology and Translational Science*.

[12] Wang Y, Engels IH, Knee DA, Nasoff M, Deveraux QL, Quon KC (2004). Synthetic lethal targeting of MYC by activation of the DR5 death receptor pathway. *Cancer Cell*.

[13] Le Meur N, Gentleman R (2008). Modeling synthetic lethality. *Genome Biology*.

[14] Nowsheen S, Cooper T, Stanley JA, Yang ES (2012). Synthetic lethal interactions between egfr and parp inhibition in human triple negative breast cancer cells. *PLoS ONE*.

[15] Barbie DA, Tamayo P, Boehm JS (2009). Systematic RNA interference reveals that oncogenic KRAS-driven cancers require TBK1. *Nature*.

[16] Dong Y, Li A, Wang J, Weber JD, Michel LS (2010). Synthetic lethality through combined notch-epidermal growth factor receptor pathway inhibition in basal-like breast cancer. *Cancer Research*.

